# Cutaneous and pulmonary cryptococcosis^☆^^☆☆^

**DOI:** 10.1016/j.abd.2019.07.009

**Published:** 2020-03-18

**Authors:** Deborah Lucena Markman, Pamella Paola Bezerra de Oliveira, Daniela Mayumi Takano, Idalina Inês Fonseca Nogueira Cambuim

**Affiliations:** a Department of Dermatology, Hospital Otávio de Freitas, Recife, PE, Brazil; b Department of Pathology, Universidade Federal de Pernambuco, Recife, PE, Brazil; c Mycology Laboratory, Hospital Otávio de Freitas, Recife, PE, Brazil

Dear Editor,

*Cryptococcus neoformans* is the etiological agent of cryptococcosis, an infectious disease that affects humans, domestic and wild animals. This pathogen is often found in pigeon excreta, possessing innumerable environmental sources.^1^

The diagnosis can be performed by analysis of cerebrospinal fluid (CSF), urine sediment, bronchoalveolar lavage fluid, wound exudates, floating node aspirate and suspected lesions biopsies.^2^

Treatment of cryptococcosis in immunocompetent and immunocompromised humans, consists of amphotericin B in combination with 5-flucitosin in disseminated infections; or with fluconazole or itraconazole, as an alternative for the treatment of cutaneous infections.^3^

A 36-year-old female patient presented an ulcerated plaque with hematic crust and erythematous infiltrated edges topped with pustules, measuring approximately 3 cm length in the posterior region of the left ear (Fig. 1). The patient related the lesion appeared 4 months before the appointment, accompanied by systemic symptoms such as intermittent fever, productive cough, and weight loss of approximately 5 kg in 15 days. Chest X-ray showed evidence of hypotransparency in the middle segment of the right lung lobe. Patient was insulin-dependent diabetic and had a history of kidney transplant performed three years ago and in use of azathioprine and prednisone. After dermatological evaluation, it was decided to perform lesional skin scrapings and mycological culture of the lesion's secretion and the sputum, as well as an incisional biopsy, with the removal of two fragments for mycological and histopathological study. Slides of direct secretion and sputum were prepared with addition of India ink upon which capsulated yeasts were observed. In the mycological culture of all the samples, there was a development of colonies of white to cream color of mucous aspect typical of *Cryptococcus* spp. The species identification and antifungal susceptibility were performed in the Vitek 2-Compact. The isolate was also identified by sequencing the D1/D2 domain of the rDNA using the primers NL1 and NL4 being identified as *Cryptococcus neoformans* (San Felice) Vuill (1901). Histopathology revealed numerous yeast fungal structures, best visualized by PAS staining (periodic acid-Schiff), with mucopolysaccharides capsules that stand out for Alcian Blue staining, with scarce inflammatory infiltrate in between (Fig. 2). The patient was hospitalized and treated with amphotericin B associated with fluconazole. After significant clinical improvement, the patient was discharged on the use of fluconazol daily until complete resolution of the cutaneous condition (Fig. 3).

**Figure 1:**
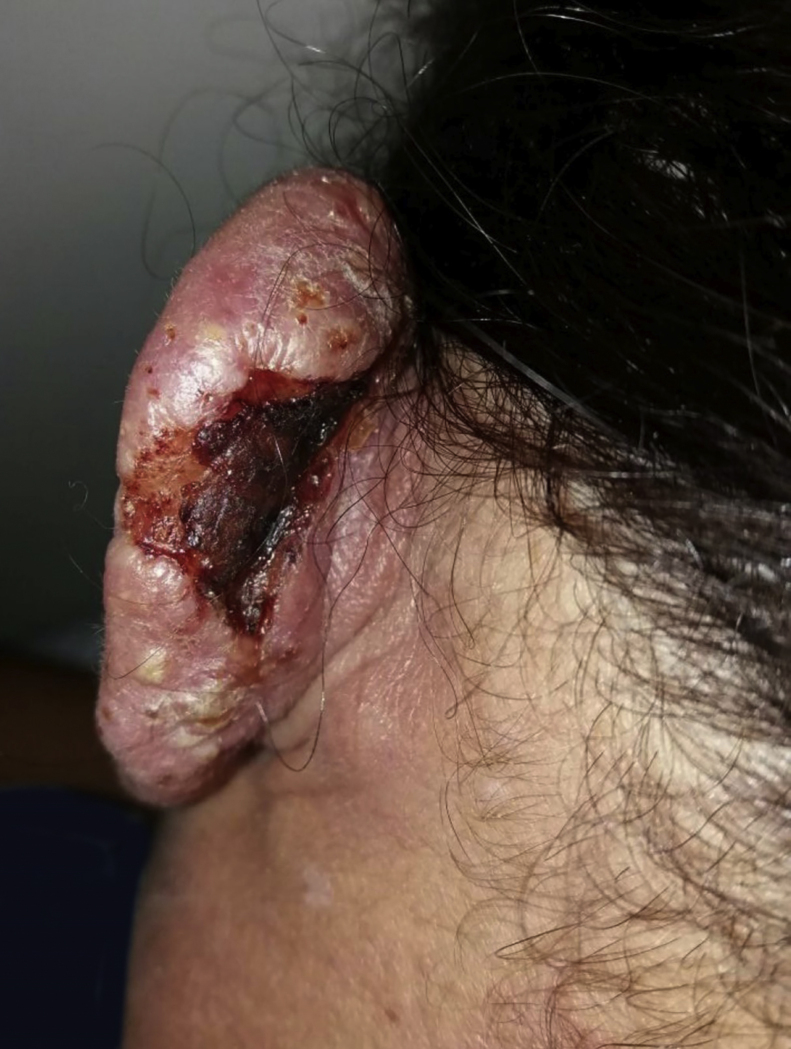
Cutaneous lesion in the posterior region of the left ear.

**Figure 2:**
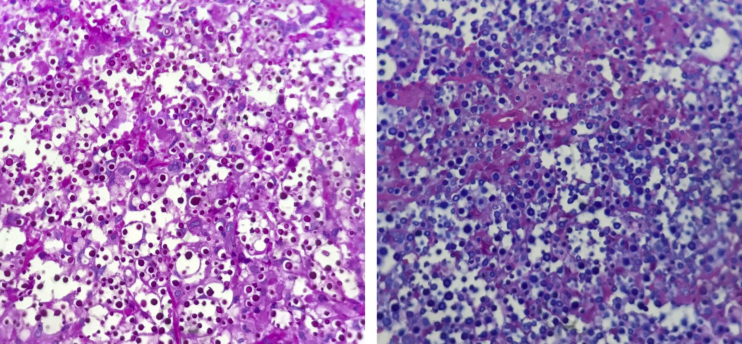
Histopathology. PAS (left) and Alcian blue (right) staining of cryptococci, ×400.

**Figure 3:**
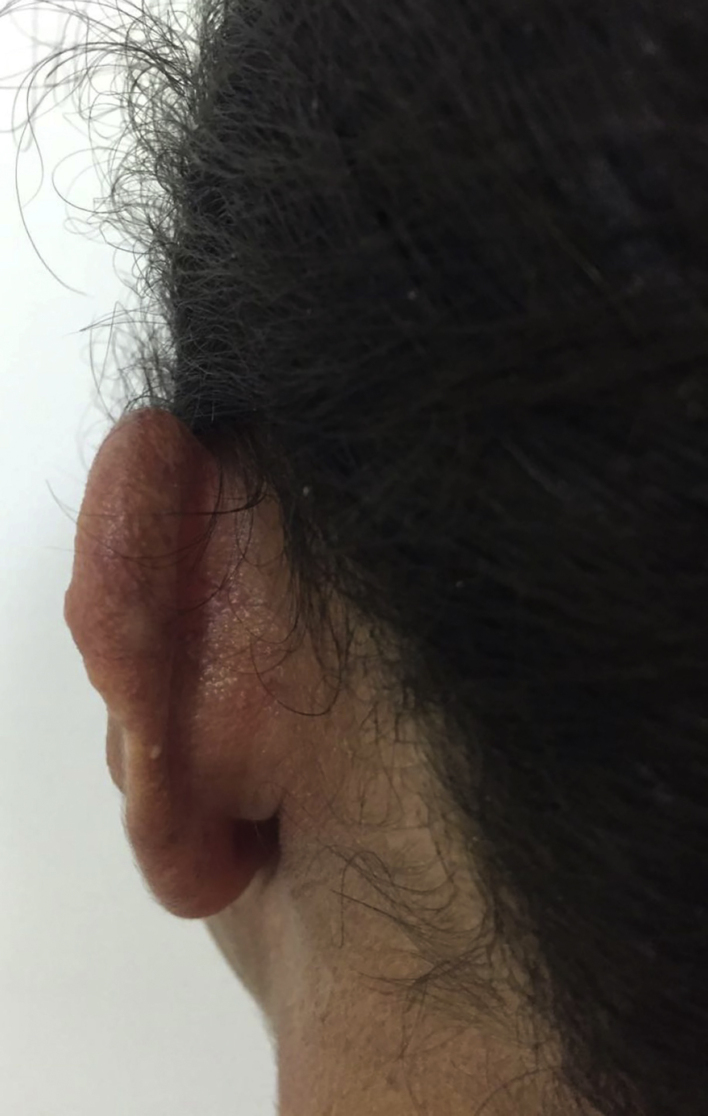
Post-treatment cutaneous lesion.

According to literature, this mycosis is commonly diagnosed in patients with cellular immunosuppression, such as HIV positive, being the most frequent systemic mycosis in this group of patients and the third cause of opportunistic Central Nervous System (CNS) disease.^1^, ^4^ In renal transplant patients, cryptococcosis occurs in 0.8–5% of them, depending on the type and intensity of immunosuppression.^5^ Infection is usually observed in the late postoperative period, about four months after the introduction of immunosuppressive drugs.^5^

Drug resistance, when using fluconazole, is likely to occur during prolonged suppressive treatments as in cases of *C. neoformans* meningitis.^1^ For the treatment of renal transplant patients using immunosuppressive drugs, drug interactions and side effects should be especially considered, particularly the intrinsic nephrotoxicity of amphotericin B.^5^ There is still no consensus regarding dose adjustment, time and duration of treatment, as well as the need of maintenance therapy.^5^

This report presents the medical history of a patient with cutaneous and pulmonary alterations, which allowed the diagnosis of cryptococcosis and the appropriate treatment for it. Through this report we could highlight the importance of the differential diagnosis of infectious conditions in renal transplant patients.

## Financial support

None declared.

## Authors’ contributions

Deborah Lucena Markman: approval of the final version of the manuscript; elaboration and writing of the manuscript; effective participation in research orientation; critical review of the literature.

Pamella Paola Bezerra de Oliveira: elaboration and writing of the manuscript; critical review of the literature.

Daniela Mayumi Takano: approval of the final version of the manuscript; effective participation in research orientation; critical review of the manuscript.

Idalina Inês Fonseca Nogueira Cambuim: approval of the final version of the manuscript; effective participation in research orientation; critical review of the manuscript.

## Conflicts of interest

None declared.
